# ESCO2 Interacts with TRF1/2 and Facilitates Telomere Maintenance

**DOI:** 10.3390/ijms27062635

**Published:** 2026-03-13

**Authors:** Jiahui Guo, Jingjing Ji, Jinfeng Liu, Mengfan Tang

**Affiliations:** 1 State Key Laboratory of Technologies for Chinese Medicine Pharmaceutical Process Control and Intelligent Manufacture, Nanjing University of Chinese Medicine, Nanjing 210023, China; 2 Department of Immunology, School of Medicine, Nanjing University of Chinese Medicine, Nanjing 210023, China

**Keywords:** ESCO2, TRF1, TRF2, telomere end protection, telomere maintenance

## Abstract

Establishment of sister chromatid cohesion N-acetyltransferase 2 (ESCO2) is an acetyltransferase involved in sister chromatid cohesion. Here we demonstrated that ESCO2 has a new role in telomere maintenance through its binding with telomeric repeat-binding factor TRF1 and TRF2. Loss of ESCO2 induces aberrant DNA damage at telomeres and leads to dramatic telomere shortening. ESCO2 associates with several proteins involved in DNA replication and repair, including BLM, WRN, TopBP1, BRIP1, BRCA1, and MUS81. Moreover, we show that ESCO2 acts in epistasis with BLM in promoting telomere stability. Taken together, our data suggest that ESCO2 is required for the maintenance of telomere stability, presumably by coordinating multiple replication and repair factors to facilitate telomere replication and protection.

## 1. Introduction

Telomeres are specialized ribonucleoprotein structures at the ends of eukaryotic chromosomes that protect linear chromosomes from erroneous recognition as DNA breaks and degradation by endogenous nucleases [1]. Telomeric DNA consists of several kilobases of double-stranded TTAGGG DNA repeats and terminates with a single-stranded 3′ overhang with a few hundred nucleotides. Telomeric DNA is assembled with a bona fide telomeric protein complex termed shelterin [2]. Shelterin is composed of six proteins: telomeric repeat-binding factor 1 (TRF1) and telomeric repeat-binding factor 2 (TRF2), which bind directly to the double-stranded portion of telomeric DNA as homodimers; protection of telomeres 1 (POT1), which binds to the telomeric 3′ overhang; and TRF1-interacting nuclear factor 2 (TIN2), repressor and activator protein 1 (RAP1), POT1- and TIN2-interacting protein (TPP1; also known as ACD), which associate with telomeres through interactions with TRF1, TRF2, and POT1 [2]. Shelterin components are crucial for protection of chromosome ends and suppressing DNA damage responses at telomeres. Researchers found that several accessory proteins bind to shelterin components and assist with proper chromosome end protection, telomere length regulation, and telomere maintenance [2,3].

To discover additional factors that participate in telomere regulation, we performed tandem affinity purification (TAP) of TRF1 and TRF2, which are constitutively present at telomeres and important for telomere maintenance. In addition to the known TRF1/2 interaction proteins, we identified the establishment of sister chromatid cohesion N-acetyltransferase 2 (ESCO2), which interacts with both TRF1 and TRF2. ESCO2 mainly functions in the establishment of sister chromatid cohesion during S phase by acetylating the cohesin component SMC3 [4]. Mutations of this gene have been associated with Roberts syndrome (RBS), with clinical features such as growth retardation, craniofacial abnormalities, and limb reduction [5]. Cellular phenotypes of RBS include lack of cohesion at the heterochromatic regions around centromeres and the long arm of the Y chromosome, reduced cell proliferation, and hypersensitivity to DNA-damaging agents [5]. In addition to its role in sister chromatid cohesion, we found that ESCO2 participates in telomere end protection and telomere stability. Loss of ESCO2 induced aberrant DNA damage at telomeres and led to significant telomere shortening, which may arise from the dramatic telomere loss that occurs during telomere replication. Furthermore, ESCO2 can interact with several DNA replication and repair factors, such as BLM, WRN, TopBP1, BRCA1, BRIP1, and MUS81, suggesting a potential role for ESCO2 in telomere protection. Additionally, we showed that ESCO2 acts in epistasis with BLM in repressing telomere replication problem. Collectively, these findings suggest that ESCO2 may maintain telomere integrity and stability by coordinating multiple DNA replication and/or repair factors to facilitate telomere replication and/or help repair of damaged telomeres.

## 2. Results

### 2.1. ESCO2 Is a Novel Telomere-Binding Protein

**Figure 1:**
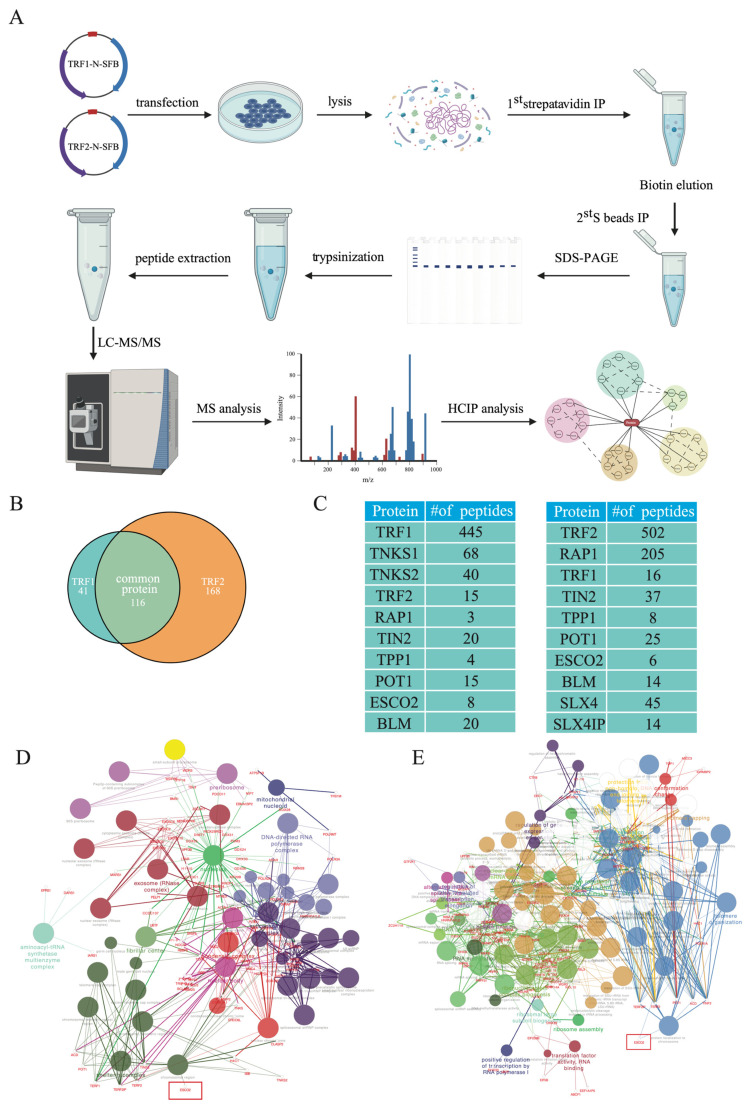
Identification of ESCO2 as a novel telomere-binding protein. (**A**) Strategy for Tandem Affinity Purification (TAP) using 293T cells stably expressing SFB-TRF1 and -TRF2. (**B**) Venn diagram showing the common and unique protein interactions of TRF1 and TRF2. TRF1 is associated with 157 interacting proteins, TRF2 with 284, and 16 proteins are shared between both. (**C**) Selected lists of TRF1 and TRF2-associated proteins analyzed by mass spectrometry. (**D**,**E**) GO pathway analysis using ClueGO in Cytoscape reveals the involvement of ESCO2 in TRF2 and its high-confidence interacting proteins. ESCO2 is highly associated with telomere-related pathways.

To uncover additional proteins that can bind to telomeres and function together with TRF1 and/or TRF2 in telomere maintenance, we performed tandem affinity purification (TAP) of TRF1 and TRF2 (Figure 1A). We employed the CRAPome database [6] to filter the observed proteins and eliminate potential false positives and ensure the identification of high-confidence interacting proteins. Ultimately, TRF1 showed 157 interactions, TRF2 had 284, and 16 interactions overlapped (Figure 1B). Mass spectrometry (MS) revealed that TRF1 pulled down not only the known TRF1-binding partners, such as tankyrases (TNKS1 and TNKS2) [7], BLM [8], and other shelterin proteins (Figure 1C and Supplemental Table S1). Similarly, TRF2 TAP-MS analysis identified shelterin proteins, BLM [8], and the SLX4-SLX4IP complex [9] (Figure 1C and Supplemental Table S1).

To further uncover these potential interactors, we conducted ClueGO Cytoscape plug-in analysis on 157 potential interaction targets of TRF1 and 284 potential interaction targets of TRF2 to identify functional groupings of the potential genes and their roles in biological processes (Figure 1D,E). This analysis also revealed highly functionally associated proteins within the identified interaction networks. Node size reflects pathway significance, and overlapping groups indicate shared functions. TRF1 interactions enriched the shelterin complex and chromosomal regions pathways. TRF2 interactions enriched telomere organization and chromosome localization pathways. A previously reported paper probed the association network surrounding TRF1, TRF2, and POT1 by using dual-tag affinity purification system to purify each telomere repeat binding factor along with their associated partners. The putative associating partners identified in their TRF1 and TRF2 TAP also participate in some biological processes such as telomere organization, DNA damage repair, chromosome cohesion, chromatin modification/remodeling, DNA replication, cell cycle and transcription regulation, nucleotide metabolism, RNA processing, and nuclear transport, which were largely consistent with our findings [10].

Notably, ESCO2 emerged as a key interactor in both pathways (Figure 1D,E). ESCO2 has not been previously linked to telomere regulation. Taken together, these findings suggest that ESCO2 probably binds to telomeres and plays roles in telomere regulation through its interaction with TRF1 and TRF2.

### 2.2. ESCO2 Interacts with TRF1/2

**Figure 2:**
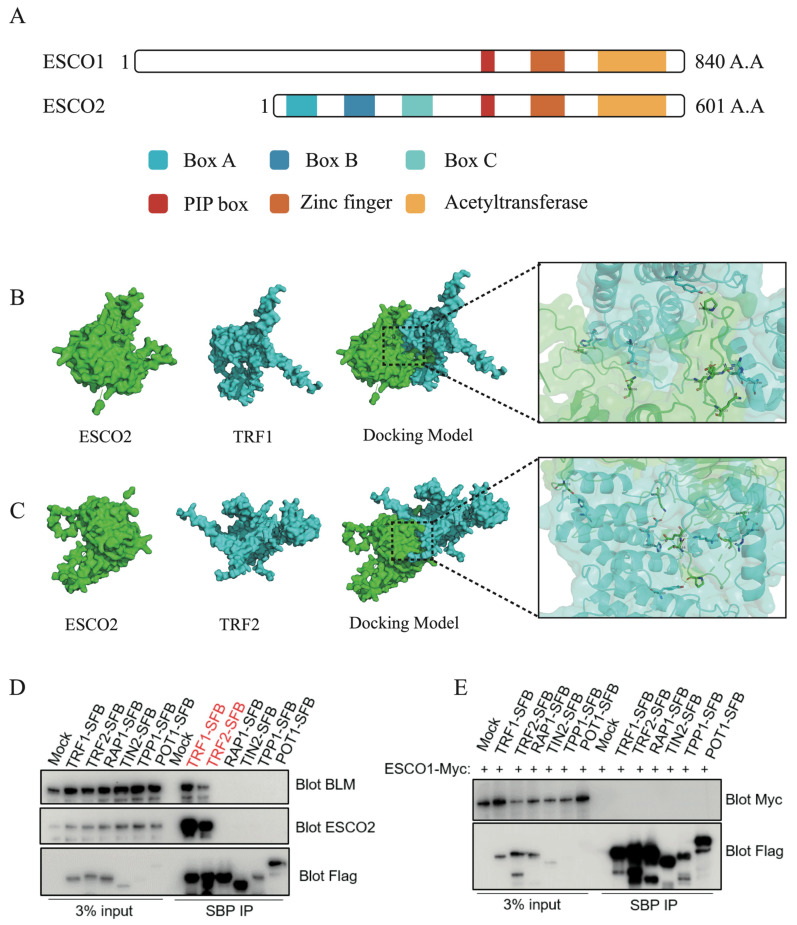
ESCO2 interacts with TRF1/2. (**A**) Scheme of human ESCO1 and ESCO2 with the conserved domains indicated. (**B**) Swiss-Model predicted 3D structure of ESCO2 and TRF1, illustrating their interaction. The structural model demonstrates the binding interface between ESCO2 and TRF1. (**C**) Swiss-Model predicted 3D structure of ESCO2 and TRF2, illustrating their interaction. The structural model demonstrates the binding interface between ESCO2 and TRF2. (**D**) Western blot showing that ESCO2 interacts with both TRF1 and TRF2. 293T cells were transfected with constructs encoding S-, Flag-, and streptavidin-binding (SFB)-tag alone (mock) or SFB-tagged shelterin proteins. Forty-eight hours later, cells were collected and lysed. Precipitation was conducted using streptavidin beads (SBP IP). Western blot was performed with the indicated antibodies. (**E**) Western blot showing that ESCO1 does not bind to shelterin proteins. 293T cells were co-transfected with constructs encoding Myc-tagged ESCO1 and SFB-tagged shelterin proteins. Forty-eight hours later, cells were collected and lysed. Precipitation was conducted using streptavidin beads. Western blot was performed with the indicated antibodies.

Unlike budding yeasts, which have only one cohesion acetyltransferase (Eco1), vertebrates express two related cohesion acetyltransferase enzymes, ESCO1 and ESCO2 [11]. ESCO1 and ESCO2 have very similar domain structures at their C-termini, which contain a PCNA-interacting protein (PIP) box, a C2H2 zinc finger motif, and an acetyltransferase catalytic domain (Figure 2A). However, ESCO1 and ESCO2 have distinct N-termini, suggesting that these two proteins have divergent functions and/or regulations in vivo. The N-terminus of ESCO2 has several short sequence elements that are important for chromatin binding [12,13,14].

To further understand the specificity of ESCO2-TRF1/2 interactions, we performed homology modeling and molecular docking using SWISS-MODEL [15]. Structures were modeled as follows: ESCO2 (UniProt: Q56NI9; template 5T53.1.A), TRF1 (P54274; 3BQo), TRF2 (Q15554; 3BU8). AutoDock Vina 1.2.5 [16] showed that ESCO2 binds to both TRF1 (ΔG = −14.3 kcal/mol) (Figure 2B) and TRF2 (ΔG = −36.9 kcal/mol) (Figure 2C). Based on the docking model and known structures, the interface involves residues from ESCO2’s N-terminal region (such as GLN206, ASP378) interacting with TRF1’s TRFH (TRF homology) dimerization domain (Figure 2B). This likely involves hydrophobic and electrostatic interactions between ESCO2’s disordered N-terminal tail and the TRF1 TRFH domain surface. Although TRF2 has a distinct N-terminal basic/GAR domain not present in TRF1, the docking model shows ESCO2 interacting with the TRFH dimerization domain which is structurally conserved between TRF1 and TRF2 (Figure 2C). In summary, the docking models show ESCO2 binding to similar surfaces on both TRF1 and TRF2 TRFH domains. ESCO2 probably uses its intrinsically disordered N-terminal region as a flexible tether to interact with multiple shelterin components, allowing it to sample different binding partners at telomeres. ESCO2 recruitment to telomeres through TRF1/TRF2 interactions may position it to acetylate cohesin and establish sister chromatid cohesion at telomeric regions during replication or other unknown functions to maintain telomere integrity and stability.

To further validate these findings, we performed co-immunoprecipitation (Co-IP) experiments to determine whether both ESCO1 and ESCO2 could interact with one or more of the six shelterin proteins. Notably, we found that, in agreement with our TAP-MS results, ESCO2 interacted strongly with TRF1 and TRF2 but not with RAP1, TPP1, POT1, or TIN2 (Figure 2D). This interaction pattern is very similar to that of BLM, which agrees with published results demonstrating interactions between BLM and TRF1 or TRF2. However, ESCO1 did not appreciably interact with any of the six shelterin proteins (Figure 2E). These data suggest that the interaction between ESCO2 and TRF1/TRF2 is very specific, implying that ESCO2 is involved in telomere maintenance.

### 2.3. ESCO2 Regulates DNA Damage Response at Telomeres

**Figure 3:**
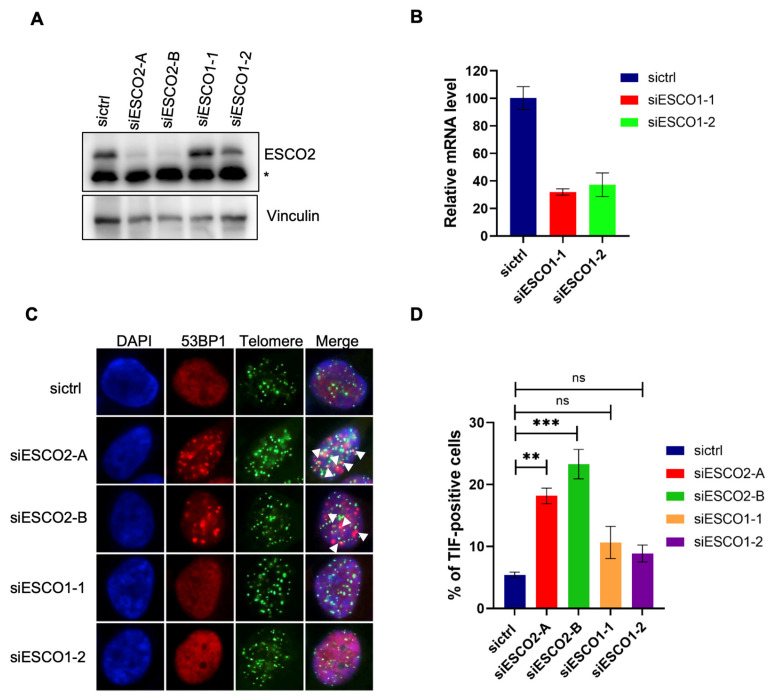
ESCO2 is required for telomere protection. (**A**) Western blot performed to determine ESCO2 siRNA-knockdown efficiency in HeLa1.2.11 cells. The * presented as the position of the non-specific band. (**B**) Quantitative real-time PCR (Q-RT-PCR) was used to examine ESCO1 siRNA-knockdown efficiency in HeLa1.2.11 cells. (**C**) Representative images of TIF analysis showing that knockdown of ESCO2 but not ESCO1 induced TIFs. HeLa1.2.11 cells transfected with the indicated siRNAs were stained with an anti-53BP1 antibody, which was followed by telomere FISH with a FITC-labeled TelC probe. DAPI was used to stain the nuclei. Arrowheads indicate co-localized foci. (**D**) Statistical quantification of the percentages of TIF-positive cells in (**C**). Cells with at least three 53BP1 foci co-localized with telomeres were considered TIF-positive cells. Data are presented as means ± standard errors (*n* = 3). ** *p* < 0.01, *** *p* < 0.001 (One-way ANOVA). ns, not significant.

Given the association of ESCO2 with telomere-associated proteins, we first examined whether ESCO2 plays a role in DNA damage response at telomeres. We transfected HeLa1.2.11 cells with respectively two different siRNAs targeting ESCO1 or ESCO2. HeLa 1.2.11 cells were specifically chosen because of their naturally extra-long telomeres which make them an ideal model for visualizing telomere signals and detecting telomere shortening via telomere fluorescence in situ hybridization (FISH). We employed Western blotting or quantitative real-time polymerase chain reaction (PCR) to confirm that both siRNAs for ESCO2 or ESCO1 worked effectively (Figure 3A,B). We then carried out immunofluorescence staining and fluorescence in situ hybridization (IF-FISH) to determine the percentage of telomere-dysfunction-induced foci (TIF)-positive cells. About 20% of cells showed TIF (indicated by foci with 53BP1 and telomere co-localization; as shown with arrowhead) in the ESCO2 knocking down group, compared with 6% in control cells and less than 10% in ESCO1-depleted cells (Figure 3C,D). These results suggested that inhibition of ESCO2 but not ESCO1 compromises the integrity of telomeres. We speculate that ESCO2 participates in telomere maintenance by regulating DNA damage response at telomeres.

### 2.4. Loss of ESCO2 Leads to Telomere Abnormalities

**Figure 4:**
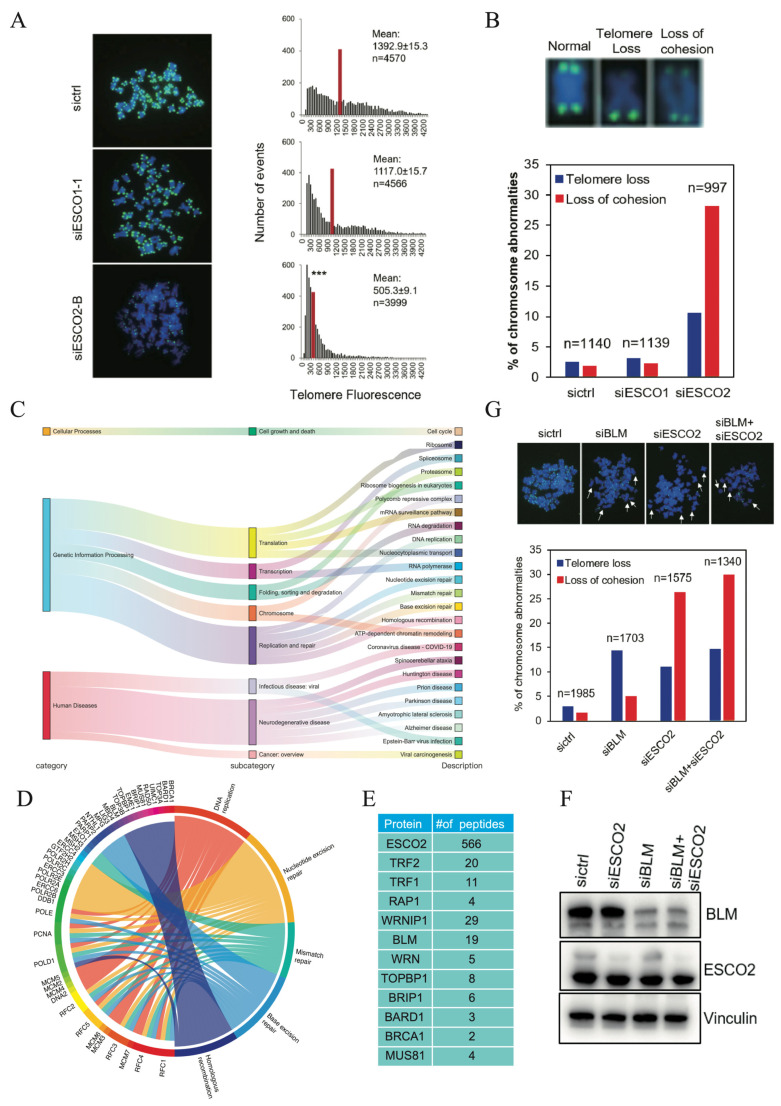
ESCO2 deficiency results in telomere abnormalities. (**A**) HeLa1.2.11 cells transfected with indicated siRNAs were analyzed by Q-FISH using a FITC-labeled telomere PNA probe. DAPI was used to stain the nuclei. The histograms show the distribution of relative telomere length presented as fluorescence intensity (TFU, telomere fluorescence unit); the red lines mark the mean telomere signal intensity. n indicates the total number of telomere signals detected. Error bars indicate standard errors. *** *p* < 0.001. (**B**) Examples of telomere abnormalities from (**A**) observed in a telomere FISH assay (upper panel). The incidence of telomere abnormalities in cells lacking ESCO1 or ESCO2 is shown in the bottom panel. (**C**) Sankey diagram showing KEGG pathway analysis of high-confidence proteins associated with ESCO2. Pathway enrichment was performed based on high-confidence ESCO2-interacting proteins, and the results are visualized as a Sankey diagram to illustrate the functional distribution across different KEGG pathways. (**D**) Chord diagram showing KEGG pathway analysis of DNA replication- and repair-related pathways and their associated proteins. Diagram visualizes the relationship between key proteins and their corresponding pathways involved in DNA replication and repair. (**E**) Selected lists of ESCO2-associated proteins analyzed by mass spectrometry. (**F**) Western blot performed to determine ESCO2 and BLM siRNA-knockdown efficiency in HeLa1.2.11 cells. (**G**) Representative images of metaphase telomere FISH in cells from (**G**) (upper panel). The incidence of telomere abnormalities is shown in the bottom panel.

We next examined whether loss of ESCO2 affects telomere length. We performed a quantitative FISH (Q-FISH) assay to measure telomere length and found that knockdown of ESCO2 but not ESCO1 significantly decreased the average telomere length (Figure 4A). The shortened telomere length may be due to frequent telomere loss. As expected, depletion of ESCO2 led to dramatically increased telomere loss, with a loss rate of 10.5% in ESCO2-depleted cells compared with 2.4% in control cells, whereas depletion of ESCO1 did not lead to a dramatic change in telomere loss (Figure 4B). We also quantified the chromosomes with loss of cohesion and found that depletion of ESCO2 resulted in loss of 30% sister chromatids cohesion, which is consistent with a previous report [17]. These data demonstrated that in addition to its role in sister chromatid cohesion, ESCO2 probably has a function in telomere maintenance and replication.

To identify the potential partners that function together with ESCO2, we performed TAP-MS analysis of ESCO2. As shown in Figure 4E and Supplemental Table S2, ESCO2 pulled down telomere proteins such as TRF1 and TRF2, which confirmed our results shown in Figure 1. Using the ClusterProfiler (version 4.0.5) R package, we performed KEGG pathway analysis on high-confidence proteins interacting with ESCO2. ESCO2 interactors enriched cell cycle, DNA replication, and repair pathways (Figure 4C). Moreover, we noticed that ESCO2 also pulled down BLM, WRN, and several other proteins involved in DNA replication and repair, including TopBP1, BRIP1, BRCA1, MUS81, and WRNIP1 (Figure 4D,E). These proteins maintain telomere stability. Their interaction with ESCO2 suggests a role in telomere replication/repair. Of note, BLM helicase is recruited by TRF1 to help break down Hoogsteen G-G base pairing in G quadruplexes at telomeres and suppress replication delays. Loss of BLM can induce increased telomere fragility and telomere loss [18,19], which are similar to the phenotypes we observed. We therefore tested whether ESCO2 would act with BLM in promoting telomere stability. We transfected HeLa 1.2.11 cells with siRNA oligos against ESCO2 and BLM either alone or combined (Figure 4F). We found that inhibition of either ESCO2 or BLM resulted in increased telomere loss but that simultaneous knockdown of both did not exacerbate telomere loss in these cells (Figure 4G), suggesting an epistasis relationship between ESCO2 and BLM in promoting telomere stability.

## 3. Discussion

ESCO2 is known to participate in the establishment of sister chromatid cohesion in S-phase cells [20]. In this study, we demonstrated that ESCO2 interacts with TRF1/TRF2 and helps the maintenance of telomere integrity and stability. Loss of ESCO2 induced aberrant DNA damage at telomeres and led to significant telomere loss, which resulted in dramatic telomere shortening. Beyond its interaction with TRF1 and TRF2, ESCO2 associates with several DNA replication and repair factors, including TopBP1, BRIP1, BRCA1, and MUS81, underscoring its broader role in coordinating telomere maintenance. Accordingly, ESCO2 appears to act in epistasis with BLM in promoting telomere stability. Collectively these findings point to a new role for mammalian ESCO2 in telomere maintenance, potentially by modulating the recruitment of multiple replication and repair factors and facilitating the resolution of telomere replication and repair.

We hypothesize that ESCO2’s role in telomere maintenance is linked to its acetyltransferase function. Acetylation is known to regulate protein interactions, stability, and function, playing a crucial role in DNA repair and replication. We propose that ESCO2 may influence the recruitment of DNA repair factors through acetylation, which, in turn, could impact telomere replication and repair. Specifically, ESCO2 may regulate telomere stability by modulating the chromatin environment and participating in stress responses related to replication at telomeres. This hypothesis warrants further experimental validation, particularly through the identification of acetylated proteins at telomeres and their functional implications.

Our findings also demonstrate that ESCO2, but not ESCO1, plays a critical role in telomere maintenance. Because the rare autosomal recessive disorder RBS is caused by mutations exclusively in ESCO2, our data imply that the defects in RBS probably caused in part by telomere abnormalities. This novel perspective provides important insights for understanding RBS pathogenesis from a telomere biology standpoint and opens potential treatment options for these patients in the clinic.

## 4. Materials and Methods

### 4.1. Cell Culture and Constructs

HeLa1.2.11 and HEK293T cells were cultured in Dulbecco’s modified Eagle’s medium with 10% fetal bovine serum in a 37 °C incubator and a 5% CO_2_ atmosphere, tested to verify negative for mycoplasma contamination. Human TRF1, TRF2, ESCO1, and ESCO2 cDNAs were cloned into a PDONOR201 vector and transferred to pBabe-CMV–based retroviral vectors for N-terminal epitope tagging with an S-, Flag-, and streptavidin-binding tag (SFB) or Myc via an LR reaction using Gateway cloning technology (Thermo Fisher Scientific, Waltham, MA, USA).

### 4.2. Antibodies, siRNAs, sgRNA

The antibodies used in this study were: 53BP1 (NB100-904; Novus Biologicals, Littleton, CO, USA), Flag (F3165; Sigma, St. Louis, MO, USA), BLM (A300-110A, Bethyl, Boston, MA, USA), ESCO2 (ab86003; Abcam, Cambridge, UK), Myc (sc-40; Santa Cruz Biotechnology, Santa Cruz, CA, USA), and vinculin (V9131; Sigma-Aldrich, St. Louis, MO, USA). The siRNAs used in this study were: sictrl (1022076; QIAGEN, Hilden, Germany), siESCO1-1, siESCO1-2 (GS114799; QIAGEN), siESCO2-A, siESCO2-B (SR315909; OriGene Technologies, Rockville, MD, USA), and siBLM-A (SR300439; OriGene Technologies).

### 4.3. Stable Cell Line Generation

HEK293T cells stably expressing TRF1-SFB, TRF2-SFB, or ESCO2-SFB were generated by transiently transfecting indicated SFB-tagged plasmids into HEK293T cells using polyethylenimine (PEI). 24 h later, cells were selected with puromycin (2 μg/mL) for 72 h until all un-transfected cells died. After replacing the cells with fresh culture medium and further culturing for two days, the stable cell lines were generated and amplified for tandem affinity purification.

### 4.4. Tandem Affinity Purification and Co-Immunoprecipitation

HEK293T cells stably expressing TRF1-SFB, TRF2-SFB, or ESCO2-SFB were harvested for tandem affinity purification. Cells were lysed in NETN buffer (20 mM Tris-HCl [pH 8.0], 1 mM EDTA, 150 mM NaCl, 0.5% NP-40, 1 mM DTT, with phosphatase and proteinase inhibitors) for 30 min and centrifuged at 16,000 rpm for 15 min at 4 °C. The supernatants were collected and incubated with streptavidin-conjugated beads (Amersham, Little Chalfont, UK) for 2 h at 4 °C. The beads were then washed with NETN buffer twice and eluted with 2 mg/mL biotin (Sigma-Aldrich, St. Louis, MO, USA) for 2 h. The eluates were incubated with S-protein beads (Novagen, Madison, WI, USA) for 2 h at 4 °C. After being washed with NETN five times, the bound proteins were resolved using SDS-PAGE and analyzed via mass spectrometry.

For co-immunoprecipitation, cells were lysed in NETN buffer (20 mM Tris-HCl [pH 8.0], 1 mM EDTA, 150 mM NaCl, 0.5% NP-40, 1 mM DTT, with phosphatase and proteinase inhibitors) for 30 min and centrifuged at 13,000 rpm for 15 min at 4 °C. The supernatants were incubated with streptavidin-conjugated beads (Amersham) for 2 h at 4 °C. After washing with NETN three times, the bound proteins were resolved using SDS-PAGE and blotted with indicated antibodies.

### 4.5. Real-Time Quantitative Reverse Transcription-PCR

Real-time quantitative reverse transcription-PCR was carried out as described previously [21]. Briefly, total RNA was isolated using TRIzol reagent (Thermo Fisher Scientific), and the same amount of RNA was used for reverse transcription with an iScript cDNA Synthesis Kit (Bio-Rad Laboratories, Hercules, CA, USA). Real-time quantitative reverse transcription-PCR amplification reactions were performed using SYBR Green PCR Master Mix (Thermo Fisher Scientific) and an ABI 7500 Real-Time PCR System (Applied Biosystems, Foster City, CA, USA). The primers used were: ESCO1 RT FP: AGAATTGGAAACACGCATGAGT; ESCO1 RT RP: GATCTCCGGTTAAGCTGTTCATT; GAPDH RT FP: ACAGTCAGCCGCATCTTCTT; and GAPDH RT RP: TTGATTTTGGAGGGATCTCG.

### 4.6. Immunofluorescence and Fluorescence In Situ Hybridization (IF-FISH)

IF-FISH for telomere dysfunction-induced foci (TIF) detection was performed as described previously and was performed with three independent biological replicates (*n* = 3) [22]. At least three 53BP1 foci co-localized with telomeres were considered as TIF-positive cells [23]. Briefly, cells harvested at 72 h after siRNA transfection were fixed for 10 min with 4% paraformaldehyde and then permeabilized for 15 min in 0.5% Triton X-100 followed by blocking with 3% bovine serum albumin for 1 h. Cells were then incubated with 53BP1 antibody (NB100-904, Novus) for 2 h at 37 °C and a secondary antibody for 1 h at room temperature. For FISH, an additional incubation step with an FITC-TelC-PNA probe (Panagene, Daejeon, Republic of Korea) was performed after secondary antibody incubation at 37 °C for 2 h. Cells were then washed twice with wash buffer 1 (10 mM Tris-HCl [pH 7.4], 70% formamide) and three times with wash buffer 2 (100 mM Tris-HCl [pH 7.4], 150 mM NaCl, 0.1% Tween 20). After dehydration with 70%-90%-100% ethanol for 5 min each, coverslips were mounted with the use of a DAPI solution (Thermo Fisher Scientific).

### 4.7. Telomere Fluorescence In Situ Hybridization (FISH)

For telomere FISH experiments, HeLa1.2.11 cells were first transfected with indicated siRNAs for 72 h and then treated with nocodazole (0.5 μg/mL) for 3 h to accumulate M-phase cells. Cells were then harvested and resuspended in 0.075 M KCl for 30 min before being fixed in methanol/acetic acid (3:1) prior to spreading. FISH was performed as described previously [24] with the use of an FITC-TelC-PNA probe (Panagene). Quantification of the average telomere length and assessment of sister chromatid cohesion were performed using the TFL-TELO program. Loss of cohesion was defined as determining the separation status of sister telomeres identified by the software. Chromosomes displaying separated sister telomere signals (exceeding the standard paired distance observed in controls) or completely parallel chromatids were scored as having lost cohesion. This quantitative approach allows for an objective assessment of cohesion defects.

### 4.8. Bioinformatics Analysis

Bioinformatics analyses were performed using RStudio (version 4.3.1), employing the ClusterProfiler (version 4.0.5) and ggplot2 (version 3.3.6) packages for functional enrichment and visualization. The ClueGO plugin (version 2.5.7) in Cytoscape (version 3.9.1) was used for Gene Ontology (GO) pathway analysis and network visualization. Mass spectrometry data were analyzed using the online database CRAPOME, with high-confidence interacting proteins defined by a SAINT score ≥ 0.9 [25] and protein foldchange ≥ 40.

### 4.9. Statistical Analysis

All key experiments were either performed as three independent biological replicates (*n* = 3) or quantified with large amounts of samples. Quantification was performed in a blinded manner (sample identities were concealed during scoring). The results were consistent across replicates. Data are plotted as means ± standard errors. Graphs were generated using GraphPad Prism 10.1, and statistical analysis was performed using one-way ANOVA or two-way ANOVA with Tukey’s multiple comparisons test and Student’s *t*-test was used for pairwise comparisons. Values of *p* < 0.05 (*), *p* < 0.01 (**), or *p* < 0.001 (***) were considered significant.

## Data Availability

The original contributions presented in this study are included in the article. Further inquiries can be directed to the corresponding authors.

## Supplementary Materials

The following supporting information can be downloaded at: https://www.mdpi.com/article/10.3390/ijms27062635/s1.
